# Novel and Unexpected Microbial Diversity in Acid Mine Drainage in Svalbard (78° N), Revealed by Culture-Independent Approaches

**DOI:** 10.3390/microorganisms3040667

**Published:** 2015-10-13

**Authors:** Antonio García-Moyano, Andreas Erling Austnes, Anders Lanzén, Elena González-Toril, Ángeles Aguilera, Lise Øvreås

**Affiliations:** 1 Department of Biology, University of Bergen, P.O. Box 7803, N-5020 Bergen, Norway; E-Mails: a.austnes@gmail.com (A.E.A.); alanzen@neiker.net (A.L.); lise.ovreas@uib.no (L.Ø.); 2 Neiker-Tecnalia, Basque Institute for Agricultural Research and Development, c/Berreaga 1, E48160 Derio, Spain; 3 Instituto de Técnica Aeroespacial, Centro de Astrobiología (CAB-CSIC), Ctra. Torrejón-Ajalvir km 4, E-28850 Madrid, Spain; E-Mails: gonzalezte@cab.inta-csic.es (E.G.-T); aguileraba@cab.inta-csic.es (A.A.)

**Keywords:** AMD, Arctic, Svalbard, acidophiles, psychrophiles, *Gallionella*, Saccharibacteria

## Abstract

Svalbard, situated in the high Arctic, is an important past and present coal mining area. Dozens of abandoned waste rock piles can be found in the proximity of Longyearbyen. This environment offers a unique opportunity for studying the biological control over the weathering of sulphide rocks at low temperatures. Although the extension and impact of acid mine drainage (AMD) in this area is known, the native microbial communities involved in this process are still scarcely studied and uncharacterized. Several abandoned mining areas were explored in the search for active AMD and a culture-independent approach was applied with samples from two different runoffs for the identification and quantification of the native microbial communities. The results obtained revealed two distinct microbial communities. One of the runoffs was more extreme with regards to pH and higher concentration of soluble iron and heavy metals. These conditions favored the development of algal-dominated microbial mats. Typical AMD microorganisms related to known iron-oxidizing bacteria (*Acidithiobacillus ferrivorans*, *Acidobacteria* and *Actinobacteria*) dominated the bacterial community although some unexpected populations related to *Chloroflexi* were also significant. No microbial mats were found in the second area. The geochemistry here showed less extreme drainage, most likely in direct contact with the ore under the waste pile. Large deposits of secondary minerals were found and the presence of iron stalks was revealed by microscopy analysis. Although typical AMD microorganisms were also detected here, the microbial community was dominated by other populations, some of them new to this type of system (Saccharibacteria, *Gallionellaceae*). These were absent or lowered in numbers the farther from the spring source and they could represent native populations involved in the oxidation of sulphide rocks within the waste rock pile. This environment appears thus as a highly interesting field of potential novelty in terms of both phylogenetic/taxonomic and functional diversity.

## 1. Introduction

Acid mine drainage (AMD) is an environmental issue caused by the weathering of metal containing minerals, normally associated with mining activities. As a result of the leaching, a solution of low pH and high concentration of dissolved metals is released into the environment, causing severe damage to the soil and vegetation [1] It is largely recognized that AMD is enhanced by the biological activity of microorganisms thriving in the acid liquors and extracting energy from the oxidation of the metals contained in the ore [2]. The microbial populations linked to this type of system are usually extremophiles, with specific adaptations to thrive in acid waters, usually highly oxidizing and normally containing elevated concentrations of heavy metals in solution. In fact, the biological oxidation of iron is the limiting step in the leaching of metallic sulphides and acid drainage formation [2]. The same microorganisms have been used with biotechnological purposes for the selective recovery of metals.

Although temperature is an obvious controlling factor in the distribution of microorganisms, in general mesophilic and moderate thermophilic acidophiles are exclusively detected in AMD [3]. It has been pointed out that the exothermic nature of the leaching, which releases heat, could be an explanation for this phenomenon [4]. In fact, no true psychrophilic acidophiles have been yet characterized in AMD, and only a few psychrotolerant strains (e.g., *Acidithiobacillus ferrivorans* and *Ferrovum myxofaciens*) have been described [3]. Moreover, since low temperature may directly or indirectly impose constraints in the overall reaction rate of pyrite oxidation [5,6] it was previously expected that the cold climates in permafrost areas (high latitude or altitude) would prevent AMD from developing. The Arctic was thus thought to be an ideal environment for mine waste disposal. This is due not only to low annual temperatures restricting biological and chemical processes, but also by the presence of a permanent permafrost layer that would lower the production and migration of pollutants. However, although the former is true, AMD occurs and it is quite extended in cold regions [1]. AMD formation in cold climates has been shown to have a markedly seasonal trend. The heat released from the leaching, keeps the liquor in liquid state, being only released upon thawing of the permafrost layer during spring [7]. Moreover, despite the temperature effect, biological activity has been detected in Arctic mine tailings at temperatures as low as −4 °C [6] and oxygen uptake due to pyrite oxidation at temperatures down to −11 °C [8]. In fact, the exothermic nature of pyrite oxidation can cause temperatures of roughly 5 °C measured at seven meters depth within a waste rock pile. This is more than 20–30 °C above ambient air temperatures [7]. Although about 60% of the heat production is related to microbial activity [9], the biological component involved in these processes is generally poorly characterized in Arctic environments [5] and it is has been suggested that unknown bioleaching bacteria may exist in these environments [6].

This is to the best of our knowledge, the first report focusing on the microbial communities associated to AMD in Svalbard, an important past and present coal mining area. Previous biological data are scarce and based on cultivation methods [7]. In this study, we have however employed a culture independent approach in order to explore the microbial communities associated with AMD in different abandoned mining areas around Longyearbyen in Svalbard, 78° N. Several areas were explored and two different runoffs were found and further analyzed with the following objectives: (i) to explore the microbial diversity associated to AMD generation in the high Arctic and (ii) to find signs of phylogenetic/taxonomic and functional novelty associated to these particular environments.

## 2. Experimental Section

### 2.1. Area Description and Sample Collection

The entire region around Longyearbyen in Svalbard is an almost continuous permafrost area, ranging typically from depths 100–500 m [10] and a mean annual temperature of −7 °C. Monthly normal values for Longyearbyen based on data from http://eklima.met.no for 2005–2015 show that just three months during the summer period have a mean temperature above 0 °C but still below 10 °C. Precipitation is approximately 250 mm annually.

**Figure 1:**
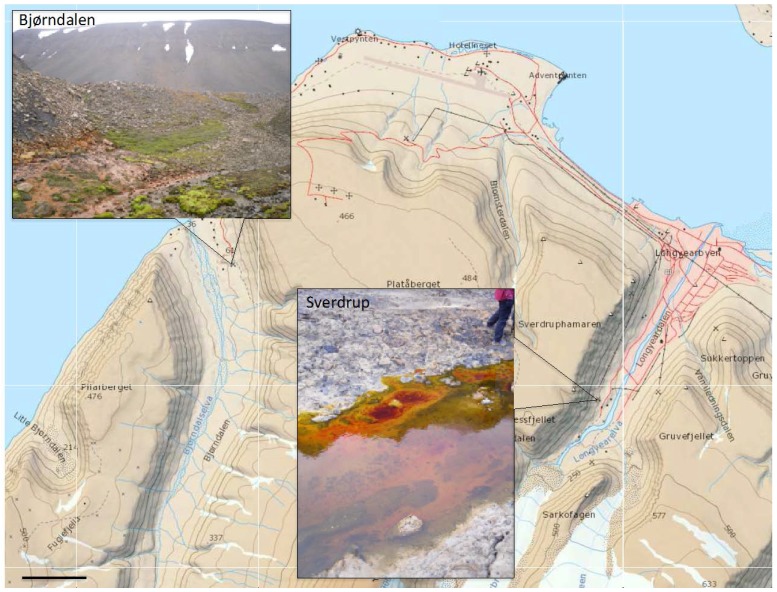
Area map that shows the study sites near Longyearbyen in Svalbard. Scale bar 1 km. Contour interval 50 m. The map was obtained from the Norwegian Polar Institute and it is licensed under the Creative Commons Attribution 4.0 International (CC BY 4.0) license (http://creativecommons.org/licenses/by/4.0/).

Sampling was carried out in August 2010 and August 2011. Up to five mine areas in the proximity to Longyearbyen (1300 km from the North Pole), including the currently operative Mine 7 were visited, in search for active acid drainage. Two runoffs were found: from a spoil tip at the abandoned Mine 3 at Bjørndalen (78°13ʹ4ʺ N, 15°19ʹ9ʺ E) and the abandoned mine at Sverdrup (78°12ʹ4ʺ N, 15°34ʹ8ʺ E). The former has previously been studied focusing on AMD generation [7,11] and its impact on the arctic vegetation [1]. A complete description of the site can be found in these studies. In summary, the waste pile is about 20 m high and consists roughly 200,000 cubic meters waste rocks deposited between 1986 and 1996, when Mine 3 was closed [1]. The discharge springs at the bottom of the pile, facing north and the surfaces along the spoil were coated with an ochre mineral phase (Figure 1). No microbial mats were observed along this stream. Samples were collected at the point where the discharge springs (Bd1) from the rock pile (Figure 1). Another nearby spring, probably permafrost thaw water was located and analyzed on site (Bd2), while further measurements and samples were taken in the intersection between both streams (Bd3 and Bd4). The spillage at Sverdrup (Sv) emerges from a spoil tip, derived from Mine 1B. It is located on the western mountainside, above the buildings of Sverdrupbyen. This mine was in operation from 1939 until 1958 when it had to close due to difficult geological conditions, although inner parts of the mine were used as a reserve of drinking water for Longyearbyen until the second half of the 1960s. Access to the mine is closed and the spring was unreachable at the time of sampling due to the amount of accumulated soft mud along the margins. Typical green-dominated (photosynthetic) massive microbial mats were observed along the leachate that run down the hillside before entering Longyear River (Figure 1) and were collected instead.

Water, sediment, and microbial mat samples were therefore collected from both areas. Water samples for geochemical analysis were filtered through a wheel filter (0.22 μm pore diameter) attached to a syringe. A duplicate sample was diluted in nitric acid for conservation purposes. Sediment and mud samples were collected with a syringe where the tip had been cut off, pulling the emboli while dipping the syringe into the substrate. The samples were then later transferred to a tube containing RNA stabilization solution. Mat samples were collected likewise or directly with a tube and also kept in RNA stabilization solution. Samples were transported to the lab facilities at UNIS (University Centre in Svalbard) where further processing including light microscopic observations and other measurements were carried out. Samples were thereafter frozen and transported to the University of Bergen for further analysis.

### 2.2. Geochemical Analyses

Water pH as well as air and water temperatures were measured on site. Dissolved metals were measured by ICP-MS (Inductively coupled plasma mass spectrometry) at the Centre for Astrobiology in Madrid. Iron speciation was determined in our laboratory by a colorimetric method [12] with a Shimadzu UV-1800 spectrophotometer (Kyoto, Japan), measuring the absorbance at 520 nm as described. A calibration curve in the range 0–2.2 mM (0–120 ppm) was fitted with a linear function (*R*^2^ = 0.99). Samples were diluted to fall within this range. Sulphate determination was based on a turbidity measurement with a saturated barium solution [13]. A calibration curve was fitted with a third degree polynomial curve over the range 0–5 mM as described.

### 2.3. Scanning Electron Microscopy (SEM)

Samples were fixed in 4% formaldehyde before different dilution aliquots were filtered through a 0.22 μm pore diameter polycarbonate filter. The preparation were dehydrated with graded series of ethanol and mounted on carbon studs before sputter coating with a gold/palladium alloy at 4.5 V for 8 s. The samples were analyzed with a Zeiss Supra 55VP (Jena, Germany) scanning electron microscope at the Electron Microscopy facilities at the University of Bergen. Elemental composition was analyzed by energy dispersive X-ray spectroscopy (EDS).

### 2.4. DNA Extraction

The ready-to-use Fast DNA SPIN kit for soil by MP Biomedicals was used with 200–500 mg (wet weight) sediment/microbial mat starting material. Different vortex speeds and times were tested, always below 5 min at maximum speed, in order to optimize maximum recovery of amplifying DNA. Extractions were always done in duplicate. DNA quality (260/280 and 260/230 ratios with spectrophotometry), quantity (fluorimetry) and integrity (DNA gel electrophoresis) were checked. 

### 2.5. RNA Gene Amplification and Cloning

Amplification of the 16S rRNA gene was carried out with specific primers. Primer set 27F (5ʹ-AGAGTTTGATCMTGGC-3ʹ) and 1492R (5ʹ-GGTTACCTTGTTACGACTT-3ʹ) [14] was used for the bacterial gene amplification while for the archaeal gene, the forward primer 20F (5ʹ-YTCCSGTTGATCCYGCSRGA-3ʹ) [15] in combination with 976R (5ʹ-YCCGGCGTTGAMTCCAATT-3ʹ) were used. The StrataClone^T™^ PCR Cloning Kit from Agilent Technologies (Santa Clara, CA, USA) was used to construct clone libraries from the 16S RNA gene amplicons. The ligated vector, with the inserted amplicon, was transformed into SoloPack^®^ competent cells (Agilent Technologies, Santa Clara, CA, USA), grown for an hour and plated onto Luria Broth ampicillin agar plates containing 2% X-Gal. After overnight incubation, randomly chosen positive transformants were directly sent for sequencing. In a different approach (see results) positive transfomants were firstly used as template for colony-PCR with M13 primers and a restriction analysis from amplified ribosomal DNA (ARDRA) was applied by quick digestion with *Taq*I (Fermentas, WA, USA) and resolved by gel electrophoresis. The banding patterns were manually compared and grouped into operational taxonomic units (OTUs). Clones with identical pattern were assumed to have identical sequence. One clone representative for each OTU was then sequenced with BigDye^®^ Terminator v3.1 (Applied Biosystems, Foster City, CA, USA) at the Centre for Astrobiology in Madrid.

### 2.6. Phylogenetic Analyses

NAST-aligned sequences were checked for chimerical assemblies with the sequence utility ChimeraSlayer [16]. Non-chimeric full-length sequences were imported into the Arb phylogenetic package [17]. The Silva SSU_Ref_104 database [18] was previously updated by including new BLASTn hits obtained from our sequences that were not already included in that database release. Multiple alignments were manually corrected with the editing tool in the software and sequences added into a stable guide tree. A distance matrix was obtained with the algorithm neighbor joining using a Felsenstein correction for DNA. The matrix was used as an input file in mothur version 1.17.0 [19]. The default algorithm furthest neighbor was chosen for cluster analysis. Rarefaction curves (1000 resampling) and a variety of community richness calculators (Chao1) and diversity indexes (Simpson) implemented in mothur were obtained. The estimated steepness values (angle theta) of the line tangent to the rarefaction were also determined by using the last point. The community structure was also analyzed in order to study the sharing level between the different samples and sites. Phylogenetic reconstruction was obtained by using the different algorithms Arb neighbour joining, PHYLIP DNAPars (Parsimony version 3.6a3, University of Washington, Seattle, WA, USA) and PHYLML (maximum likelihood version 2.4.5) implemented in the Arb software (Technical University of Munich, Munich, Germany). Only sequences >1000 nucleotides were used. Filters excluding the most variable positions were also employed. A consensus tree was selected among the generated trees by comparing the stability of the branching. Only one representative of each OTU was finally kept on the tree for better understanding. Sequences of unique phylotypes found in this study have been deposited on GenBank under accession numbers LN880450 to LN880512.

### 2.7. qPCR

Total 16S rRNA copy number was determined by qPCR with group-specific primers for *Bacteria*, *Archaea* and known iron-oxidizing bacteria (*Gallionella* spp., *Sideroxydans* spp. and *Ferrovum myxofaciens*). Bacterial 16S rDNA genes were quantified by using the primers 338F (5ʹ-ACTCCTACGGGAGGCAGC-3ʹ) and 519R (5ʹ-TTACCGCGGCKGCTG-3ʹ) as described before [20]. The primers Arch349F (5′-GYGCASCAGKCGMGAAW-3′) and Arch806R (5′-GGACTACVSGGGTATCTAAT-3′) were used for archaeal rDNA [21]. Each 20 μL-reaction contained 1× QuantiTech Sybr Green PCR master mixture (Qiagen, Valencia, CA, USA), 0.5 mM of each primer, and 1 to 10 ng of template DNA. Thermal cycling was programmed as follows: 95 °C for 15 min + 30 × (94 °C for 15 s + 61 °C for 30 s + 72 °C for 30 s) + melting curve (50–95 °C). The quantification standard was run in triplicate and consisted of a dilution series of a purified 16S rDNA containing-plasmid DNA. The standard ranged typically from 10^2^ to 10^8^ copies/μL. All samples were run in duplicate and different DNA extractions and template concentrations were employed. A blank (no template DNA) and a negative control (no target DNA) were always introduced for each run. Product specificity and eventual primer dimer formation were checked for on the melting curve and by gel electrophoresis. The standard curve was generated using at least five concentrations within the dilution series. The *R*^2^ value for the standard curve was typically >0.99 and slope values indicated efficiencies between 95% and 105%. All qPCR experiments were conducted in a Step-OnePlus real-time PCR system (Applied Biosystems). Relative copy number of *Gallionella*/*Sideroxydans* and *Ferrovum* 16S rDNA genes were performed likewise by using the newly designed primer Gal217F (5ʹ-TCGCTTTCGGAGTGGCC-3ʹ) and the already described primer [22] Gal384R (5ʹ-GGTATGGCTGGATCAGGC-3ʹ). The *Ferrovum*-specific primers Fer643F (5ʹ-ACTGGCAAGCTAGAGTCTGT-3ʹ) and Fer847R (5ʹ-TCGCGTTAGCTTCGTTACTGA-3ʹ) were employed [23]. Annealing temperatures for these primers were optimized at 60 °C and 63 °C respectively. The standard consisted of a dilution series of a purified 16S rDNA carrying plasmid obtained from Bjørndalen (for *Gallionella*/*Sideroxydans*) and Rio Tinto (for *Ferrovum*). As above, the standard curve as generated using at least five concentrations within the dilution series. The slope of the fitted linear function (*R*^2^ > 0.99) gave a estimated amplification efficiency between 97% and 106%.

Gene copy numbers were used to calculate the estimated percentage of the bacterial community of single iron-oxidizing groups by including the specific 16S rRNA copy per genome for each group. These were obtained from the rrnDB database version 4.3.3 [24]: 3.99 for *Bacteria*, 2.5 for *Gallionella*/*Sideroxydans* and 1 for *Ferrovum myxofaciens* [25].

### 2.8. Amplicon Library Construction and 454 Sequencing

The V5–V8 region of prokaryotic SSU rRNA (16S) was PCR amplified from extracted DNA using the primers Uni787F (5ʹ-ATTAGATACCCNGGTAG-3ʹ) and Uni1492RM (5ʹ-GNTACCTTGTTACGACTT-3ʹ) [26] using a two-step (nested) PCR protocol described previously [27]. The first PCR was optimized for each sample by finding the DNA extraction, template concentration and cycling conditions that provide maximum efficiency in the shortest possible number of cycles and equal concentration of final products. In order to do this, templates obtained from different extraction conditions, template concentrations and number of PCR cycles were monitored for each sample by means of qPCR. The final conditions used for the construction of the amplicon library, which had given >95% efficiency amplification were as follows: 8–10 ng of template DNA was cycled 25 times (average C_t_ value of 18.4 for all samples). Triplicate PCR reactions for each sample were pooled and purified using GenElute PCR Clean-Up kit (Sigma, Oslo, Norway) prior to the second five-cycle PCR step to construct the amplicons with a barcode (“multiplex identifiers”) and GS-FLX adaptors (Lib-L). Resulting amplicons were cleaned using AMPure XP (Beckman Coulter, Oslo, Norway) following the manufacturer’s protocol (bead-to-sample ratio 9:10). Amplicon DNA was analyzed using gel electrophoresis to ensure complete removal of primers and negligible amounts of non- barcoded product. Concentrations were measured using fluorometry (Qbit, Thermofisher Scientific, Waltham, MA, USA) and amplicons stored at −80 °C until pooling in equimolar amounts and sequencing. Pyrosequencing were performed at the Norwegian High-Throughput Sequencing Centre in Oslo using Lib-L chemistry and GS-FLX Titanium technology (454 Life Science, Roche, Penzberg, Germany). The raw data (SFF files) have been submitted to the European Nucleotide Archive with study accession number PRJEB9921.

### 2.9. Noise Removal and Taxonomic Clustering

Filtering and removal chimera sequences were performed using AmpliconNoise [28]. The resulting denoised sequences were clustered into OTUs using a 3% distance cut-off and rarefied. Diversity indexes (Simpson 1-D and Shannon Hʹ) were also calculated. Finally, reads were subjected to taxonomic classification using Classification Resources for Environmental Sequence Tags CREST [29].

## 3. Results

### 3.1. Observations and Chemical Analysis

The chemical parameters from both areas showed some significant differences (Table 1). The leachate at Sverdrup shows a typical chemistry from pyrite-rich spoils. The water pH (mean 2.4) and the iron and sulphate concentrations are indicative of intense pyrite weathering thus leaching other metals, which appear also in elevated concentration, e.g., Al (76 ppm), Zn (5.1 ppm) Ni (1.3 ppm), Mg (145 ppm) or Mn (9.7 ppm). Most of the parameters at Bjørndalen are typical of acid drainage, although somewhat less toxic containing less concentration of some metal ions. There were, however, some peculiarities including the interesting observation that the vast majority of iron in solution is in reduced rather the oxidized state. Actually, most of the iron, and possibly other metals appear as a solid phase, coating the substrate, despite the low pH (mean 3.1) (Figure 1). In fact, the molar ratio of sulphate to iron is almost 7:1, two times higher than in Sverdrup. The water that springs under the spoil is the most aggressive, but few meters downstream it merges another source of fresh water, probably supra-permafrost summer thaw water (pH 6.2). This mixed water (Bd3) has an average pH 3.6 although and the concentration of most metals is significantly diluted: *ca*. 2 ppm Fe and 30 ppm sulphate (Table 1). Both streams flow for a few meters before merging other water sources and finally the main river running through the valley.

### 3.2. Clone Libraries and Phylogeny

Amplification of Bacteria-specific sequences was obtained from all samples. A total of 48 clones were directly sequenced from each of the Bjørndalen clone libraries Bd1 and Bd3. These retrieved respectively 36 and 34 de-noised and nearly full 16S rRNA gene sequences. Cluster analysis produced respectively 17 and 26 OTUs, based on a 3% cut-off value. A similar amount of clones were also directly sequenced from a library produced from Sv1 sample. However 98% of these retrieved plastid-related 16S rRNA sequences according to BLASTn. An ARDRA-based filtering gave a better resolution (see Material and Methods). More than 200 insert-bearing clones from the Sv2 sample were then analyzed by ARDRA and these retrieved 36 OTUs based on their restriction pattern. After sequencing, these were further clustered in 14 OTUs. Rarefaction curves for both libraries show a different trend (not shown). Sv2 approaches a plateau (θ < 0.7°) while the Bjørndalen libraries are still far from saturation although they show a different progression (θ = 12° for Bd1 and θ = 35° for Bd3). Even with a sampling effort as big as the smallest of the Bjørndalen libraries, 26 clones, the steepness value for the tangent line that crosses the best-fitted polynomial function for the Sverdrup curve, has a θ = 5.2°. This is considerably different from the steepness at the same point on both Bjørndalen’s curves. This indicates that besides the effect from a different sampling effort, there is a significant difference with regards to the diversity in both libraries.

**Table 1:** Physico-chemical parameters measured on site. The table shows the average values for pH and concentration of different metals measured in the water at each location. Iron speciation determined by colorimetric methods is also shown. All values are expressed in mg·L^−1^. Na means not analyzed.

Area	Bjørndalen				Sverdrup
Samples	Bd1	Bd2	Bd3	Bd4	Sv1, Sv2
Type	Spring water	Thaw water	Mix upstream	Mix downstream	Mat and sediment
pH	2.8	6.2	3.5	3.8	2.3
°C (water)	9	7	7	7	4
°C (air)	6	6	6	6	4
Na	3.1	2.5	3	2.8	42
K	1.1	0.5	1.6	1.3	0.2
Zn	0.3	Na	0.1	0.07	5.1
Ni	0.1	Na	0.03	0.02	1.3
Mg	5.4	1.3	2.6	2.3	145
Ca	Na	Na	Na	Na	10
Mn	0.4	Na	0.2	0.1	9.7
Cu	0.03	Na	Na	Na	0.8
Al	3.4	Na	1.8	0.9	76
Fe total	15	Na	2	2	883
Fe^2+^	9	Na	1	1	183
Fe^3+^	6	Na	1	1	700
SO_4_^2−^	110	10	54	32	1543

**Figure A1:**
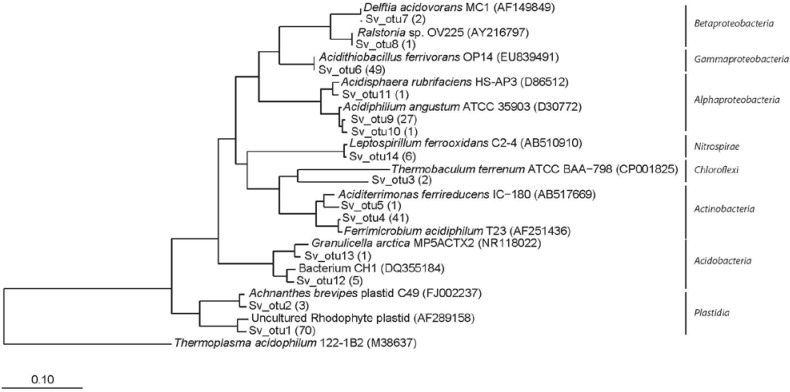
Phylogenetic affiliation for the 16S rRNA gene sequences obtained from the Sverdrup library (Sv). Representative clones for different phylotypes from mat and sediment were selected. A maximum parsimony tree was chosen as a consensus tree after reconstructing the phylogeny by using different algorithms, substitutions models, and filters. Related isolates or environmental sequences are shown for comparison purposes. Bar represents 10% estimated phylogenetic divergence.

**Figure A2:**
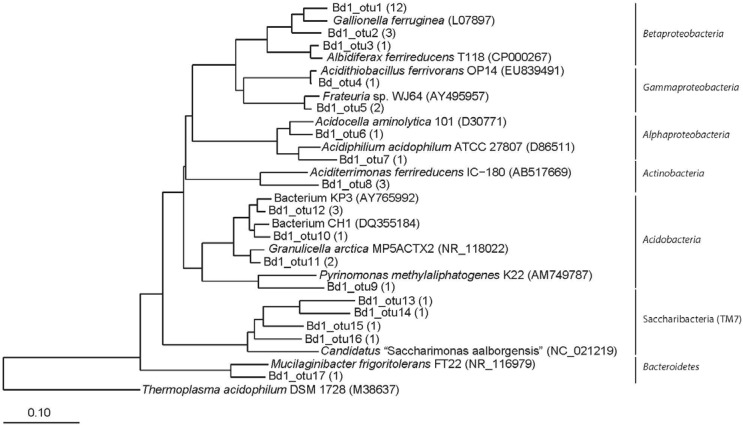
Phylogenetic affiliation for the 16S rRNA gene sequences obtained from the Bjørndalen library (Bd1). Representative clones for different phylotypes were selected, retrieved from the sediment and water springing from the waste pile. A maximum parsimony tree was chosen as a consensus tree after reconstructing the phylogeny by using different algorithms, substitutions models, and filters. Related isolates or environmental sequences are shown for comparison purposes. Bar represents 10% estimated phylogenetic divergence.

**Figure A3:**
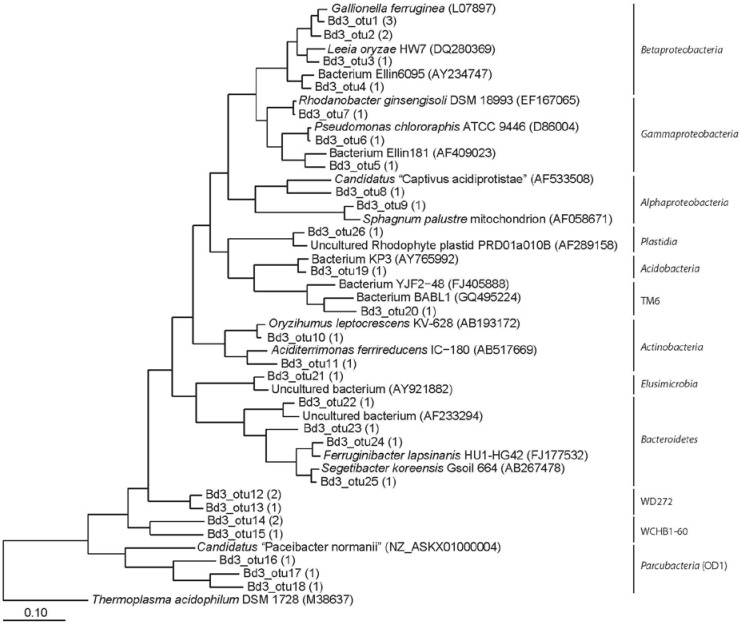
Phylogenetic affiliation for 16S rRNA gene sequences obtained from the Bjørndalen library (Bd3). Representative clones for different phylotypes were selected, retrieved from the sediment and water downstream the spring site. A maximum parsimony tree was chosen as a consensus tree after reconstructing the phylogeny by using different algorithms, substitutions models, and filters. Related isolates or environmental sequences are shown for comparison purposes. Bar represents 10% estimated phylogenetic divergence.

Taxonomic assignment and phylogenetic analysis from the Sverdrup library revealed a low phylum-diversity (six main phyla) similar to Bd1 library (five phyla/candidate divisions) but considerable small compared to Bd3 (10 phyla/candidate divisions). Their phylogenetic position is represented in supplementary Figure A1, Figure A2 and Figure A3 Sequences obtained from Sverdrup were derived from organisms that phylogenetically related to the *Proteobacteria*. Within the Alpha class, several OTUs were found related to the genera *Acidiphilium* and *Acidisphaera* (both within the family *Acetobacteraceae*). Within the Beta class, two OTUs were related to *Ralstonia* and *Delftia* (*Burkholderiales*). All the OTUs within the Gamma class were closely related to the psychrotolerant iron oxidizing bacterium *At. ferrivorans*. All the actinobacterial OTUs clustered within the *Acidimicrobiaceae*, closely related to the genera *Ferrimicrobium* and *Aciditerrimonas*. One single OTU clustered within the phylum *Chloroflexi*, with no close cultivated representative. Some sequences were related to the iron oxidizing bacterium *Leptospirillum* (phylum *Nitrospirae*) and several other OTUs to different members of the *Acidobacteria* like *Granunicella* or the unclassified acidobacterium CH1. Finally sequences clustering the *Cyanobacteria*, and most likely representing photosynthetic plastidial sequences from both *Rhodophyta* and *Bacillariophyceae* (diatoms) were also retrieved.

The phylum *Proteobacteria* was also represented in the Bjørndalen libraries (Figure A2 and Figure A3). The Bd1 library contained some OTUs within the Alpha class related to the acidophilic heterotrophs *Acidocella* and *Acidiphilium* (*Acetobacteraceae*). The Beta class was represented by few OTUs related to *Gallionellaceae* in addition to an OTU related to *Albidiferax ferrireducens*. The acidophilic *At. ferrivorans* (Gamma class) was also detected in this library together with the acid thriving *Frateuria* spp. The phylum *Actinobacteria* was also represented with an OTU related to the iron reducing bacterium *Aciditerrimonas*. The *Acidobacteria* were also well represented, with few OTUs related to *Pyrinomonas*, *Granulicella*, and *Acidobacterium-*like. One more OTU clustered with the genus *Mucilaginibacter* within the phylum *Bacteroidetes*. Finally, it was surprising the amount of OTUs that clustered the candidate divisions Saccharibacteria (formerly TM7). The library Bd3 showed some similarities but also showed more diversity in terms of OTU richness. Sequences related to *Gallionellaceae* (*Betaproteobacteria*) were also retrieved here. Some other genera and unclassified soil bacteria from the Beta- and Gamma- classes were also detected here. The *Alphaproteobacteria* class were represented by some mitochondrial sequences and the symbiont Candidate “Captivus acidiprotistae”. OTUs within the phylum *Actinobacteria* (*Aciditerrimonas* and *Oryzihumus*) and unclassified members of the phylum *Acidobacteria* were also detected. Several OTUs represented different genera within the *Bacteroidetes*, such as *Ferruginibacter*, *Segetibacter* and some uncultured members. One OTU was clearly related to *Rhodophyta* plastidial ribosomic sequences. Also one single OTU clustered tentatively with the *Elusimicrobia*. The high diversity of candidate divisions including “Parcubacteria” (OD1), WCHB1-60, WD272, or TM6 was also surprising.

**Figure A4:**
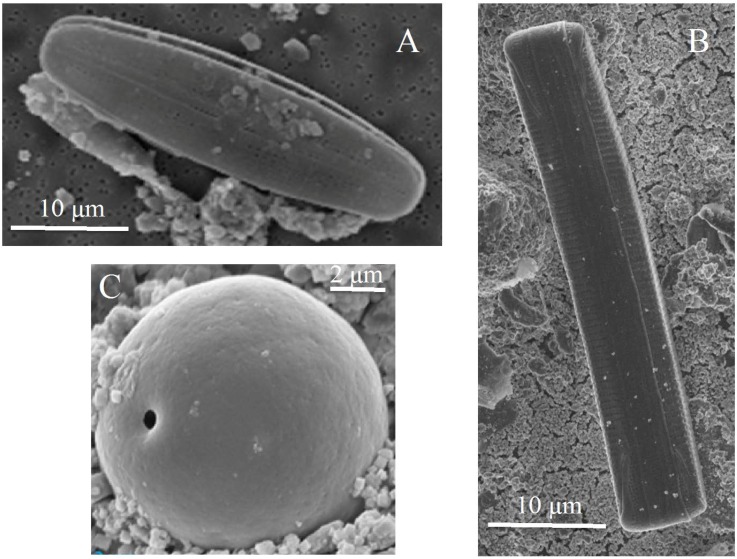
Scanning electron micrographs of two pennate diatoms (**A,B**) observed in the samples from Sverdrup as well as an eukaryote with physical similarities to Chrysophyceaen stomatocysts (**C**).

No amplicons were obtained from any of the samples when the archaeal primers were used, although positive controls never failed. The presence of eukaryotes was evident in the Sverdrup area, and their presence was confirmed by microscopic observations (Figure A4) and further extended through mitochondrial, plastidial, and other symbiont-related sequences. No massive microbial mats were observed at Bjørndalen and no eukaryotic organisms were detected under the microscope from the spring site (Bd1). Moreover no eukaryotic sequences were obtained from this spot, neither in the clone libraries nor the tagged amplicon library. However the samples collected downstream from the spring at the Bjørndalen site (Bd3) produced some sequences after PCR with eukaryote-specific primers. Ciliates (*Orthamphisiella*, *Lacrymaria*), yeasts (*Leucosporidium*), chlorophytes (*Chlorella*) and some other unclassified eukaryotes from a contaminated aquifer were retrieved (accession numbers).

When the clone libraries from both sites were compared, only five OTUs (at 3% cut-off value) were shared between them. This represents 8% of the joint observed richness (48 OTUs). These OTUs were assigned to *At. ferrivorans*, *Aciditerrimonas* spp. the acidobacteria CH1 and KP3 and an unclassified rhodophyte.

### 3.3. Community Composition and Diversity

**Figure 2:**
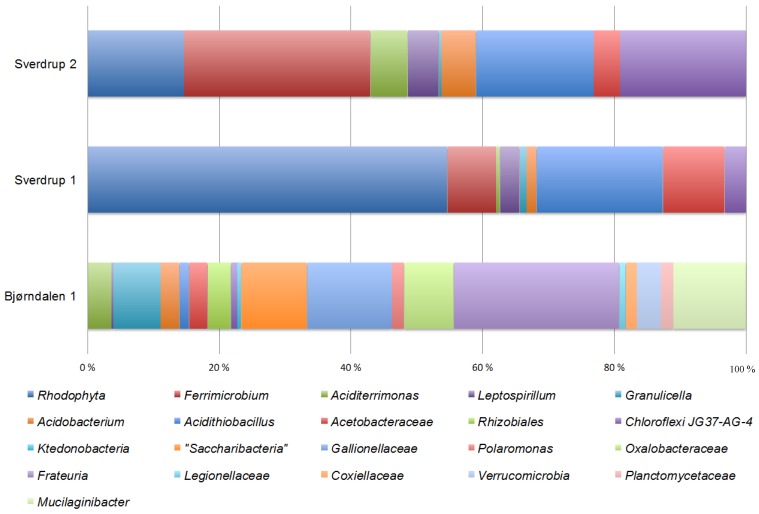
Microbial community composition on the different samples from acid mine drainage at Bjørndalen and Sverdrup areas. The figure shows the percentage of classified reads from the tagged amplicon library for each sample.

Richness amounted to 3569 OTU (3% distance) in Bd1 *versus* 72–80 in the Sverdrup samples. Estimated diversity indexes predict a higher diversity in Bjørndalen compared to Sverdrup (Table A1). Figure 2 shows the taxonomic clustering of OTUs for both sites. It is remarkable the difference in taxa richness between both sites. The microbial community at Bjørndalen shows a high diversity, with a total of 2209 OTUs from 18,324 quality sequences assigned to nine different bacterial phyla: *Proteobacteria* (*Alphaproteobacteria*, 7%; *Betaproteobacteria*, 15%; *Gammaproteobacteria* (19%), Saccharibacteria (23%), *Actinobacteria* (8%), *Bacteroidetes* (8%), *Acidobacteria* (7%), *Verrucomicrobia* (6%), *Chloroflexi* (4%), *Planctomycetes* (2%), and *Nitrospirae* (1%). On the other hand, the microbial community at Sverdrup has in comparison a very low diversity. More than 15,000 filtered sequences clustered in only 90–100 OTUs and were assigned to five phyla: *Rhodophyta*, *Actinobacteria*, *Proteobacteria* (*Alphaproteobacteria* and *Gammaproteobacteria*), *Acidobacteria*, *Chloroflexi*, and *Nitrospirae*. Figure 2 also shows the difference in community composition in Sverdrup between two mat/sediment samples. No significant differences in the overall diversity between samples but only differences in the relative abundance of some taxa. More than 50% of the reads from the Sv1 were related to plastid sequences from *Rhodophyta*. This corroborates our results obtained with the clone libraries and validates our ARDRA-based approach.

The pyrosequencing results are in accordance with the 16S rRNA gene clone libraries. However, compared to those, a higher level of similarity was now found between both sites with regards to their community composition. Shared OTUs were related to *Acidithiobacillus*, some *Actinobacteria* (*Aciditerrimonas*), *Acidobacteria* (*Granunicella* and other *Acidobacteraceae*), as well as some *Acetobacteraceae* (*Acidiphilium*). Remarkable differences in community composition between both sites were also found. OTUs affiliated to *Frateuria*, *Gallionellaceae*, and Saccharibacteria were preferentially obtained at Bjørndalen, while affiliations to *Ferrimicrobium* and the phylum *Chloroflexi* were exclusive to the Sverdrup samples. Pyrosequencing also failed to detect any abundant archaeal members.

## 4. Discussion

More than 40 abandoned coalmine rock piles can be found around Longyearbyen [7]. This extension represents an enormous potential for microbial communities involved in the weathering of sulphide rocks and AMD generation. Moreover some factors like the rock and mineral composition, the age (mining activity on Svalbard spans more than 100 years), hydrology, *etc.* might account for significant local differences in between these sites. Seven different areas were explored in this survey and two active AMD runoffs were located. The time of the year probably precluded more runoffs to be found. The water table was likely at its lowest, coinciding the minimum annual precipitation in August, although it probably fluctuates and reaches its maximum upon thawing in spring [1]. The fact that the source emerges at a lower altitude (125 m *vs*. 175 m above sea level) and that the mine was used as a reserve of drinking water, might account for the larger runoff in Sverdrup compared to Bjørndalen. This larger volume develops into more toxic drainage and facilitates the development of typical photosynthetic-dominated biofilms growing over the loose sediment [30]. This transition has been observed for other systems [31]. The increase in levels of oxygen released by photosynthesis enhances the oxidizing reactions and lead to an increase in ferric iron [31] by iron-oxidizing microorganisms such as *Acidithiobacillus*, *Leptospirillum*, or *Ferrimicrobium*. Photosynthetic biofilms at Sverdrup seemed to have no problem capturing light, probably due to the shallow water but also the moderate concentration of ferric iron. It has been shown that the hydrolysis and precipitation of ferric iron leads to a decrease in pH and the resulting iron oxide deposition might eventually inhibit algal development [32]. Both the tagged amplicon and the clone libraries show a diversity-poor bacterial community at this site, dominated by typical AMD microorganisms, involved in both the iron and sulphur cycles. Although further and extended specific quantification is required to circumvent primer bias and PCR drift, heterotrophic populations seem to represent a high percentage of the community (*Actinobacteria*, *Acidobacteria*, and *Acetobacteraceae*) pointing again towards an active primary production driven by light. Actually our first attempt to build a clone library resulted in a mask-effect due to the amount of plastid-related sequences. Even the tagged-amplicon library shows that up to 55% of the community is eukaryotic, mainly photosynthetic. Many of the heterotrophic populations are known to be very versatile, playing a role in facilitating growth not only of chemolithotrophic microorganisms (by biodegrading toxic organic compounds) but also for the photosynthetic algae (by solubilizing the iron deposits that might inhibit their development). Although intriguing, the presence of *Chloroflexi*-related phylotypes is not new to AMD systems [33,34,35]. Yet their role in the microbial community is still unknown, a recent genomic analysis across this phylum finds no evidence for a role in sulphur cycling and indicates instead roles in sediment carbon cycling [36]. This group might represent a significant portion of the microbial community at Sverdrup. In fact, most of the *Chloroflexi*-related reads clustered within one single OTU, which might indicate an important role for these microorganisms.

On the other hand, both the geochemistry and the biological data indicate that the water springing at Bjørndalen (Bd1) is most likely in direct contact with the ore within the rock pile and represents a unique window to observe the microbial communities associated with this environment. Although a more detailed geochemical analysis is required, the water springing at Bd1 was mainly reducing and its geochemistry changed quickly when in contact with the atmosphere (Table 1) and especially when mixed with other surface running water sources. Water pH and metal concentrations were most extreme at Bd1 which correlated well with the lower diversity obtained at Bd3 with the clone libraries. More complex bacterial communities tend to be found in moderately acidic pH in comparison with low pH communities [37,38,39]. Many typical AMD-related microorganisms are also detected at Bd3 (Figure A3). However the clone libraries included other phylotypes, many of them uncultured representatives from candidate divisions. It is remarkable the presence of OTUs related to the candidate division OD1 (“Parcubacteria”), which have mainly been detected in sulphur-rich, anoxic sediments and have been suggested to have a role in anaerobic methane oxidation [40]. Some phylotypes were related to the uncultured candidate division WD272 for which the genome of *Candidatus* “Fodinabacter communificans” has been reconstructed from a metagenomics project on organisms from an arsenic-rich acid mine drainage, and it has been suggested to play a crucial role in the microbial community [41]. Some other clones (division WCHB1-60) showed similarity to sequences retrieved from an anaerobic, chlorinated, and hydrocarbon contaminated aquifer [42]. Moreover, members of the globally distributed yet uncultivated candidate phylum TM6 were also detected. The genome sequence of one member within this phylum points as well towards a facultative anaerobic metabolism [43]. In fact, it is surprising the number of microorganisms that are allegedly symbionts or obligate community members [43,44,45,46,47]. Moreover some of these microorganisms detected at Bd3 are likely native to Arctic tundra soils and seasonal melt water [48,49,50,51] and some others have even been found associated to sulphide rock weathering in Antarctica [52]. Due to this difficulty of discerning between native AMD populations and populations introduced by the soil cover, we decided to focus our efforts into Bd1 for a better in-depth analysis. Many of the populations detected at Bd1 are known facultative anaerobic, able to carry out dissimilatory reduction of ferric iron (e.g., *At.* *ferrivorans*, *Albidiferax ferrireducens*, *Acidiphilium* sp., *Acidocella* sp., *Aciditerrimonas ferrireducens*, *Frateuria* sp. [53,54,55,56] as well as known micro-aerophilic iron-oxidizing microorganisms (*Gallionellaceae*). This composition points again to a more reducing environment underneath the rock pile, favorable for the development of anaerobic or micro-aerophilic metabolisms. Moreover, the water temperature measure at Bd1 was slightly higher, possibly due to active leaching and heat release within the pile as previously described [1]. The diversity revealed both by the tagged-amplicon and clone libraries were again in agreement. As in other mine tailings environments previously studied, the microbial community at Bjørndalen is dominated by members from Bacteria, both with regards to diversity and biomass, while Archaea and Eukarya were present in low abundance and therefore might play a minor role [57]. In fact, while some few eukaryotic sequences were retrieved, no archaeal sequences were detected by any of the PCR-based techniques (Table A2). The community at this spot was made of typical acidophilic microorganisms linked to the iron (*At. ferrivorans*, *Aciditerrimonas*, *Acidobacteria*, *Frateuria*, *Acetobacteraceae, Gallionellaceae*) and sulphur cycles (*At. ferrivorans*) suggesting an importance of transformation processes within these two biogeochemical cycles. It is particularly interesting the amount of different OTUs related to the recently proposed Candidate phylum Saccharibacteria (formerly TM7). This intra-phylum diversity is also reflected in the tagged amplicon library: more than 20 different OTUs were defined, representing up to 23% of the total library reads (Figure 2). Further quantification by qPCR or hybridization techniques is needed to confirm this observation. Although little is known about the metabolic traits of Saccharibacteria, it is recognized that some members of this phylum favor acidic soils with high metal content [58]. In fact the phylum seems to have a site-specific nature, with very few cosmopolitan members [47]. These two premises together with their relative abundance and the fact that they were only detected in Bd1, indicate an important role in the microbial community within the rock pile, maybe involved in the active leaching. 

**Figure A5:**
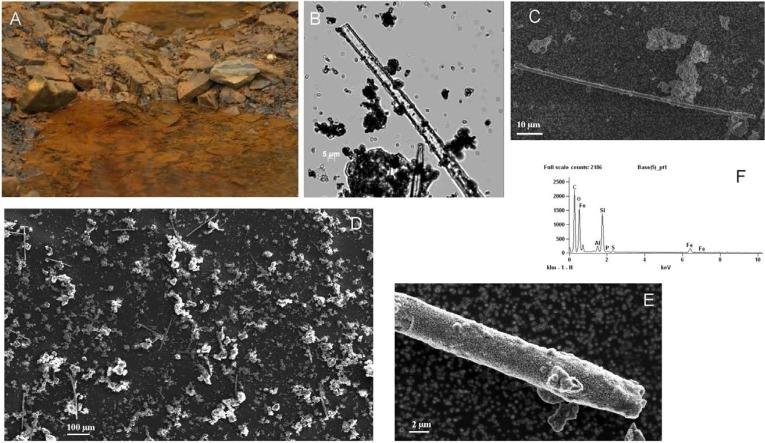
(**A**) Close up picture of the ochre mineral coat at Bjørndalen. (**B**) Phase contrast light micrograph showing iron stalks. (**C**,**D**) Scanning electron micrographs of iron stalks. (**E**) Energy dispersive X-ray spectroscopy (EDS) coupled to scanning electron microscopy from an iron stalk.

**Figure A6:**
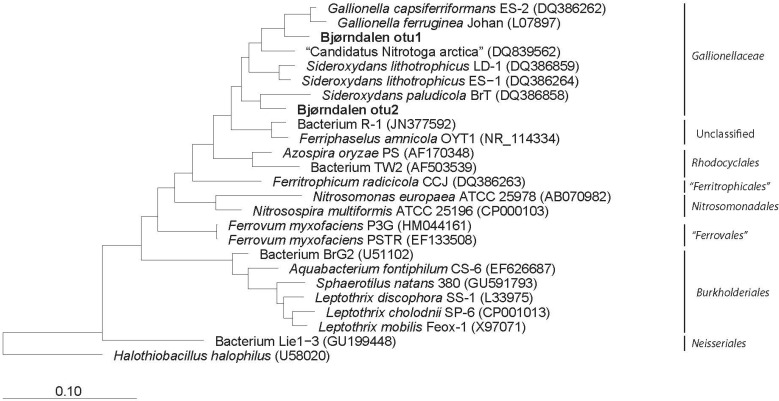
Maximum-likelihood tree showing the phylogenetic relationship between some known iron oxidizing bacteria and other bacteria within the *Betaproteobacteria* class. The relative position of the two different *Gallionellaceae* phylotypes retrieved from Bjørndalen are shown. The scale bar represents 10% nucleotide substitutions.

However, it is true that specific quantification of the *Gallionella*/*Sideroxydans* genes showed a similar abundance of these organisms to that obtained with the tagged amplicon library (Table A2). *Gallionella*-like sequences have previously been obtained from acidic environments leading to the idea that some members within this group of bacteria exhibit a greater acid and metal tolerance than previously believed [25,59]. Most of the reports concerning *Gallionella*/*Sideroxydans* in acidic environments failed however to detect the typical twisted stalks and it was until recently generally accepted that stalk formation does not start below pH 6 [60]. A recent report however describes the surprising abundance of *Gallionella*-related iron oxidizers in acidic (pH 4.4) sediments as well as the presence of typical twisted stalks [25]. In our study we detected non-twisted iron stalks (Figure A5) more similar to the sheaths described for *Leptothrix*, another known iron-oxidizer [61] although they were wider in average (about 2 μm) than those. While only one major OTU within the *Gallionellaceae* comprises all the reads in the tagged amplicon library from Bd1, two different OTUs (94% identical between them) are clearly defined in the clone libraries from both Bd1 and Bd3, providing a better resolution for their taxonomic and phylogenetic placement. In fact, while one of these two OTUs show highest identity (95%) to *Gallionella ferruginea*, the second one is most similar (95%) to *Sideroxydans paludicola* BrT. This is further supported by their phylogenetic position (Figure A6). Moreover, this phylogeny places *Sideroxydans paludicola* BrT separated from *S. lithotrophicus*, consistent with previous works [62,63] and points to an urgent revision of this genus. Sequence identity and phylogenetic distance to other known iron-oxidizers within the *Betaproteobacteria* class (including *Leptothrix*) ranges 88%–94%. The identity of these two putative species remains therefore unknown until further isolation and characterization, as well as their role in the formation of Fe-oxide biogenic structures. It is remarkable however, that no strains of *Sideroxydans* have been shown to produce twisted iron stalks, and that this phylotype is also the less abundant of the two *Gallionellaceae* detected in the clone library from Bd1 (Figure A2). One way or another it is particularly interesting the fact that many of the *Gallionellaceae* sequences associated to acidic environments, normally occur at a low (5–17 °C) temperature [64,65,66,67] and are also associated with primary production and soil fertility in glacial ecosystems [68,69,70]. Even in temperate latitude areas, *Gallionellaceae* seem to flourish during the cold winter months [71,72]. These findings reinforce the interest in AMD in cold areas as a potential niche for new psychrophilic or psychrotolerant acidophiles. It is however intriguing that no signs of the known psychrotolerant [73] iron-oxidizing bacterium *Ferrovum myxofaciens* were found or detected, not even with specific primers, neither at Bjørndalen nor Sverdrup areas. The absence of this microorganism is also consistent with the lack of typical acid streamers and floating microbial filamentous mats [74] where this bacterium tends to flourish. One explanation could be their acidic susceptibility and their preference for relatively high ferrous iron conditions [23].

Although further studies are needed to confirm their nature, origin and formation conditions, this study represents the first report of biogenic iron oxide structures at pH below 3. In fact, one of the major questions to answer is whether these sheaths are native to the sediment within the acid runoff or were originally formed under different conditions within the rock pile and later deposited upon thawing of the permafrost layer in spring and the flush of the winter-accumulated drainage [1]. In fact, most of the iron at this spot is not kept in the solution (15 ppm total soluble iron) but rather deposited in large quantities of secondary iron phases near the pile, visible mainly as ochre on the ground and vegetation (Figure 1 and Figure A5(A)) above the actual water level. The poor crystalline properties of these iron oxides hindered their diffraction analysis and were not determined, although sea-urchin morphologies typical from schwertmannite were also observed [75]. It is however known that temperature affects the equilibrium of iron mineral precipitates [5,76]. The identification of these secondary minerals might help to solve some these questions. The study of the water in contact with the ore, especially during the occluded pile during the winter months should therefore be a future priority. Moreover it has been documented that heat generation within the rock pile due to active leaching, is high enough to keep the pile at roughly 5 ± 1 °C throughout the year [1] and that oxidation can continue to temperatures as low as −11 °C [77]. Despite these premises, no true psychrophilic acidophiles are known but only psychrotolerant species have been described so long. The temperature might in fact be an important constraining factor in this system. Biological activity (iron oxidation) is hindered when temperature drops [5]. However it is also known that certain metabolic reactions become thermodynamically more favorable as temperature decreases [78], indicating not only the taxonomic and functional novelty but also the biotechnological potential of these communities in comparison to other systems already described in temperate areas. Climate regime and seasonality might impose an environmental constraint, which is translated into a specialized and novel microbial community, responsible for the mineralization of sulphide minerals in polar latitudes. This novelty implies that the biological control in acid drainage generation could be different to what it is known for other temperate areas and that some of the diversity detected in the High Arctic might be linked to unique metabolic processes.

## 5. Conclusions

This environment appears as a highly interesting field of potential novelty in terms of both phylogenetic/taxonomic and functional diversity. The detection of acidophiles with new physiological traits allows for the further development of novel strategies for the remediation of this important pollution problem, extremely detrimental to the environment, which is also crucial to the exploitation of mineral resources worldwide. Knowledge about the native microbial communities controlling the weathering is therefore essential to evaluate the impact on the ecosystem (soil acidity, toxic metal release, vegetation damage) and make appropriate decisions about the remediation and management. This is particularly relevant for the Arctic region, a sensitive area where the rock is actively exposed to weathering due to ongoing mining activity.

## Appendix

**Table A1:** Overview of sequence datasets.

Site name	PCR Cycles *	Raw reads	Filtered reads	% Chimeric reads	Unique reads	3% OTUs	Chao1 (3%)	(1−D) **	*H*ʹ **	Rarefied Richness
Bd1	25	21,698	18,324	1.33%	3693	2209	3569	0.98	6.06	2 034
Sv1	25	17,409	15,368	0.02%	92	72	116	0.83	2.11	65.9
Sv2	25	17,716	15,670	0.05%	100	80	152	0.67	1.69	73.2

* PCR cycles used for preparation of amplicon libraries. ** Shannon (*H*ʹ) and Simpson (1−D) diversity indices estimated from the distribution of 3% OTUs for amplicon datasets.

**Table A2:** Enumeration of Bacteria, Archaea, and the iron oxidizers *Gallionella*/*Sideroxydans* and *Ferrovum* 16S rDNA copies for the AMD samples collected at Bjørndalen and Sverdrup using qPCR. Copy numbers are given per gram of sample material (wet weight). Total bacterial cell numbers were calculated assuming an average number of 4.3 copies per cell. For *Gallionella*/*Sideroxydans*, cells per gram were divided for one as the genome sequences deposited contain an average of one copy.

Sample	Archaeal 16S rDNA Copies	Average Bacterial 16S rDNA Copies	*Gallionella*/*Sideroxydans* 16S rDNA Copies	*Ferrovum* 16S rDNA Copies	Estimated Bacterial Cells per Gram *	*Gallionella*/*Sideroxydans* % Copies of Total
Bd1	not detected	3.27 × 10^8^	4.49 × 10^7^ ± 9.7%	not detected	3.8 × 10^7^ ± 0.4%	13.7% ± 1.32
Bd3	not detected	1.62 × 10^8^	8.82 × 10^6^ ± 37%	not detected	1.9 × 10^7^ ± 31%	5.5% ± 2.03
Sv1	not detected	5.27 × 10^7^	2.31 × 10^4^ ± 3.3%	not detected	6.1 × 10^6^ ± 28%	<1% ± 0.03
Sv2	not detected	2.64 × 10^8^	2.30 × 10^6^ ± 9.9%	not detected	3.06 × 10^7^ ± 5%	<1% ± 0.1

* Estimated bacterial cells per gram of wet sediment assuming a100% efficiency in the DNA extraction.
